# High-grade synovitis associates with clinical markers and response to therapy in chronic inflammatory arthritis: *post hoc* analysis of a synovial biomarkers prospective cohort study

**DOI:** 10.3389/fimmu.2023.1298583

**Published:** 2024-01-11

**Authors:** Carlo Garaffoni, Marianna Tamussin, Ilaria Calciolari, Giovanni Lanza, Alessandra Bortoluzzi, Carlo Alberto Scirè, Marcello Govoni, Ettore Silvagni

**Affiliations:** ^1^ Rheumatology Unit, Department of Medical Sciences, University of Ferrara and Azienda Ospedaliero-Universitaria S. Anna, Ferrara, Italy; ^2^ Anatomic Pathology, Department of Translational Medicine, University of Ferrara, Ferrara, Italy; ^3^ Istituti di Ricovero e Cura a Carattere Scientifico (IRCCS) San Gerardo dei Tintori Foundation, Monza, Italy; ^4^ School of Medicine, University of Milano Bicocca, Milan, Italy

**Keywords:** rheumatoid arthritis, psoriatic arthritis, chronic synovitis, Krenn’s synovitis score, response to therapy

## Abstract

**Background:**

Inflammatory arthritis (IAs), such as rheumatoid arthritis (RA) and psoriatic arthritis (PsA), are characterized by the presence of chronic synovitis. The Krenn’s synovitis score (KSS), a simple tool detectable by haematoxylin/eosin staining of synovial biopsy samples, allows the discrimination between high-grade and low-grade synovitis. The aim of this study was to identify the clinical associations of KSS and to evaluate the relationship between high-grade synovitis and treatment response in IA patients.

**Methods:**

Clinical, laboratory and ultrasound data were retrieved from RA and PsA patients recruited in the prospective MATRIX cohort study. Inclusion criteria were age≥18 years, RA or PsA diagnosis, and presence of active disease with eligibility to start/modify therapy. Patients underwent ultrasound-guided synovial biopsy of one of the most involved joints before starting/modifying treatment according to treat-to-target strategy. The samples were analysed by an expert pathologist for KSS calculation. Univariable and multivariable logistic regression analyses were performed to evaluate the relationship between KSS and baseline variables. The association between KSS and treatment response at 24 weeks of follow-up was investigated in univariable logistic regression analysis.

**Results:**

53 patients, 34 RA and 19 PsA, completed 24 weeks of follow-up after synovial biopsy. Patients were either treatment naïve (N=6, 11%), csDMARDs-experienced (N=46, 87%) or b/tsDMARDs-experienced (N=20, 38%). Median KSS was 6.00 (Q1-Q3 4.00-7.00) in RA and 4.00 (3.00-6.00) in PsA (p=0.040), and inflammatory infiltrates score was significantly higher in RA than in PsA patients (median 3.00 vs 2.00, p=0.021). In multivariable analysis, synovial effusion in the biopsied joint (OR 9.26, 95%CI 2.12-53.91) and erythrocyte sedimentation rate (ESR) (OR 1.04, 95%CI 1.01-1.08) associated with high KSS. High-grade synovitis significantly associated with a higher probability of achieving DAS28 remission, ACR20/50 response, and Boolean2.0 remission, independently from diagnosis.

**Conclusion:**

Several markers of pro-inflammatory pathways associated with the presence of high-grade synovitis, and patients with higher KSS shared a higher probability of treatment targets achievement in the follow up. The integration of a simple and feasible tool like KSS in the clinical and prognostic stratification of patients with IA might help in intercepting patients with a disease more prone to respond to available treatment paradigms.

## 1 Introduction

Chronic inflammatory arthritis (IAs) encompass a variety of diagnostic entities, including rheumatoid arthritis (RA) and psoriatic arthritis (PsA), all characterised by the presence of chronic synovitis, sharing wide within-disease heterogeneity and between-disease common pathogenic processes (1, 2). When inadequately treated or refractory to standard treatments, IAs may lead to disability, impairment of health-related quality of life, and an increase in mortality. To date, treating rheumatologists have access to several effective disease-modifying anti-rheumatic drugs (DMARDs) approved for RA and PsA (conventional synthetic (cs), biological (b), and targeted synthetic (ts) DMARDs) (3, 4). Most treatments are efficacious in different diseases, but still have a relevant proportion of failures, or suboptimal responses (5, 6). Therefore, matching the right drug to the right patient at the right time, though desirable, is a challenging task in chronic IAs treatment. Actual treatment guidelines recommend a stepwise approach in the management, starting from csDMARDs, and moving to b/tsDMARDs, with not yet available validated predictive biomarkers of response (7–9). To this end, clinical, serological and imaging biomarkers have been extensively investigated to optimise outcomes, without achieving satisfactory results so far (10–12).

Synovial membrane is thought to be the primary target of inflammation in RA, and one of the primary targets in PsA, and studies on synovial biomarkers have been undertaken to evaluate the early effects of different drugs and to predict treatment response (13, 14). At present, reduction in CD68-positive macrophages of the sublining (sl) represents the most accepted and validated response biomarker in longitudinal studies in RA, applicable to several types of drugs, while the reduction in CD3+/CD68+sl cells applies to tumour necrosis factor-alpha inhibitors (TNFis) treatment in PsA (13, 15). Relevant advances in the study of synovial membrane in RA have helped in dissecting the heterogeneity of RA synovium, with different pathotypes stratified across patients and phases of the disease (16–23). However, synovial membrane analysis has not yet entered clinical practice to help clinicians in driving therapeutic decisions, and this aspect remains one of the major unmet needs in the field of chronic inflammatory arthritis, with treatment decisions remaining mainly based on the ‘heuristic’ “trials-and-errors” approach (24–26).

Of particular importance in the reporting of the outcome of synovial biopsy examinations, the Krenn’s Synovitis Score (KSS) is a semi-quantitative histopathological scale which, by means of hematoxylin-eosin staining of the synovial tissue samples obtained through biopsy, allows the quantification of the degree of inflammation (27–29). Synovial lining hyperplasia, stromal cell density and inflammatory infiltrate are the three components that are quantified (0 to 3) and added together to obtain the total KSS score (0 to 9). A total score greater than or equal to 5 indicates the presence of high-grade inflammatory synovitis. Although the use of a semi-quantitative synovitis score is suggested by EULAR (European Alliance of Associations for Rheumatology) and OMERACT (Outcome Measures in Rheumatology) (30), to date few studies evaluated the association between KSS and clinical variables in patients with chronic IAs (31, 32). Event though the KSS is a simple, feasible and informative score for describing the inflammatory burden of synovitis, its association with clinical and instrumental patients’ factors, as well as its role in predicting response to DMARDs and guiding therapeutic decisions, remain uncertain (22). Therefore, we conducted this prospective study to analyze (i) the clinical and instrumental variables associated with high-grade KSS and inflammatory infiltrates; (ii) the relation between high-grade KSS and treatment response; and (iii) the association between synovial infiltrate and treatment response in a cohort of IA patients.

## 2 Materials and methods

### 2.1 Ethics approval, patients and public involvement

The study protocol was approved by the Ethics Committee of Area Vasta Emilia Centro-Emilia-Romagna (*698/2020/Sper/AOUFe*, approval 05/08/2020). All procedures were in accordance with the ethical standards of the responsible committee on human experimentation and with the Declaration of Helsinki. All subjects provided signed informed consent and were asked to assess the burden of the intervention.

### 2.2 Study design and setting

The MATRIX study is a prospective, experimental, multicentre, diagnostic accuracy study. Here, we provided data from a *post hoc* analysis assessing the prognostic role of KSS.

Patients with IA attending the Rheumatology Unit, Department of Medical Sciences, University of Ferrara and Azienda Ospedaliero - Universitaria S.Anna, Cona (FE), meeting the following inclusion criteria, were eligible for this study: (i) confirmed diagnosis of RA (33) or PsA (34); (ii) age greater than or equal to 18 years; (iii) active peripheral synovitis; (iv) potential indication to start/modify therapy; (v) ultrasound-guided synovial biopsy of one of the most involved synovial joints; (v) availability of 24-week follow up data; (vi) written informed consent provided. Exclusion criteria were: (i) contraindication to start/modify therapy; (ii) contraindication to synovial biopsy; (iii) treatment with intra-articular steroids within the previous month; (iv) patients with dementia or an altered mental state that would have precluded the understanding and rendering of informed consent.

### 2.3 Study procedures

Ultrasound-guided synovial biopsy was performed at the Rheumatology Unit, Department of Medical Sciences, University of Ferrara and Azienda Ospedaliero - Unversitaria S.Anna, Cona (FE), as part of a comprehensive clinical assessment of active IA patients (‘Refractory Arthritis Clinic’), encompassing clinical evaluation, musculoskeletal ultrasound assessment, joint radiographic assessment, and synovial histopathological evaluation, scored according to KSS for chronic synovitis (27). After 15 days from the ultrasound-guided synovial biopsy procedure, patients started/modified DMARDs therapy following the therapeutic indications of the treating rheumatologist, according to international recommendations (7, 9) and regular clinical practice.

Data captured only at basal visit included:

- Demographic variables (gender, date of birth, ethnicity);
- Lifestyles: smoking habits, alcohol use, physical activity, body mass index (BMI);
- Medical history: comorbidities;
- Disease classification according to EULAR/American College of Rheumatology (ACR) criteria for RA (33), CASPAR criteria for PsA (34) and date of diagnosis; Moll and Wright subgroups (35), serological status (rheumatoid factor, RF, and anti-citrullinated protein antibodies, ACPAs);
- Global OMERACT–EULAR Synovitis Score (GLOESS) for RA patients (36)
- Previous treatment history;
- Patient’s pain, stiffness and swelling of the biopsied joint (0-100).

At baseline and follow up visit (24 weeks), a standard clinical assessment was performed, in order to evaluate clinical response, included ACR response (37), Disease Activity Score CRP (DAS28-CRP) response (38) and ACR/EULAR Remission Criteria 2.0 (39), including:

- Health Assessment Questionnaire–Disability Index (HAQ-DI) (40);
- Patient global activity (PGA);
- Global Health (GH);
- Global pain visual analogic scale (VAS Pain);
- Physician global assessment (PhGA);
- Joint count;
- Disease Activity in PSoriatic Arthritis (DAPSA) Score (41);
- Ultrasound assessment of the biopsied joint (42);
- Erythrocytes sedimentation rate (ESR) and C-reactive protein (CRP);
- Ongoing treatment and treatment modification;
- Adverse events (AEs).

Ultrasound examination was performed by experienced rheumatologists and musculoskeletal ultrasonographers (CG, CS, ES), using a commercially available real-time scanner (3-18 MHz, Samsung RS80A). The ultrasound assessment of the joint suitable for ultrasound-guided synovial biopsy was scored according to Global OMERACT–EULAR Synovitis Score (GLOESS) score for synovitis (36), considering effusion, synovial hypertrophy and Power Doppler signal. For all the ultrasound examinations, the procedures were performed at room temperature. Smoking or use of nicotine substitutions 12 hours before the examination were not permitted.

Ultrasound-guided biopsies were performed with a 14G guillotine-type biopsy-needle (Precisa 1410-HS or Precisa 1415-HS, Hospital Service Spa) for the knee joint, or a 18G guillotine-type biopsy-needle (Precisa 1810-HS, Hospital Service Spa) for the wrist joint, according to recommended procedures (30, 43–45). During each procedure, 6-to-8 fragments were retrieved per joint to guarantee an adequate material for the histological analysis.

Data were recorded using a secure electronic data capture database for IA patients (https://www.redcap.ospfe.it) (46), hosted at the University Hospital of Ferrara.

### 2.4 Histology analysis

Histopathological evaluation was scored by a single expert pathologist (GL) according to KSS (27). Tissue samples were fixed in formalin for 24h, embedded in paraffin and stained with haematoxylin/eosin for routine histology. Histopathological evaluation was performed according to the pathologist’s experience and scored accordingly, following KSS, for (i) enlargement of the synovial lining cell layer, (ii) density of the resident cells, (iii) inflammatory infiltrate (**Supplementary Table S1**). At least two sequential sections for each patient were evaluated, and the highest score obtained was recorded. Globally, the sum of the values for each parameter was summarized as follows: a score of 0–1 (no synovitis), 2–4 (low-grade synovitis), and 5–9 (high-grade synovitis). The pathologist (GL) was unaware of the patients’ clinical and immunologic characteristics.

### 2.5 Statistical analysis

The results of the descriptive analyses were presented as mean and standard deviation (SD) or median and interquartile range (Q1-Q3) for continuous variables, according to their distribution. Qualitative variables were reported as frequencies and percentages. T test or non-parametric Wilcoxon rank sum test were used for descriptive analysis of continuous variables, assessing the normality of the variable distributions with graphical inspection of histograms and Q-Q plots. The comparison between-group of qualitative variables was performed with Chi-square test or Fisher’s exact test. The association between high-grade synovitis (KSS≥5) and relevant clinical variables was assessed in an exploratory univariable logistic regression analysis. Then, variables with significance level <0.10 were included in multivariable model and the selection of covariates was conducted with the backward stepwise method. The same analysis was repeated considering as dependent variable the presence of Krenn’s inflammatory infiltrates score equal to three. Univariable logistic regression models for prediction of response to therapy were fitted with high-grade synovitis and Krenn’s infiltrates as explanatory variables. Response to therapy was defined as DAS28-CRP<2.6, Boolean2.0 remission criteria, ACR20 or ACR50 response criteria (24 weeks). Finally, multivariable models for prediction of response were fitted by adding diagnosis as a covariate. The statistical analyses and the generation of the graphs were performed using RStudio^©^ software (47).

## 3 Results

### 3.1 Baseline characteristics

53 patients (34 RA and 19 PsA) who underwent ultrasound-guided synovial biopsy were enrolled between 14^th^ September 2020 and 1^st^ September 2022. The synovial biopsy was performed in 20 right knees, 31 left knees and 2 left wrists. The procedure was well tolerated and only 2 patients reported minor AEs (one patient had lipothymia during the procedure and one experienced an acute worsening of the arthritis in the biopsied joint during the week after the biopsy). **Table 1** shows the baseline characteristics of IAs patients included. RA patients were more frequently female (76% vs 32%, p=0.003), and they had a lower average weight (mean ± SD 71 ±16 Vs 81 ±13, p=0.034) and higher mean ESR value compared to PsA patients (42 ±25 Vs 26 ±25, p=0.020). 13/34 (38.2%) RA patients had seronegative disease and 17/34 (50%) had double positivity for RF and ACPA. Patients with moderate or severe disease activity, calculated as DAS28-CRP, were 48/53 (90.4% of patients); using the other definitions, 98.1% of the patients had moderate-to-severe disease activity according to SDAI and 96.2% to CDAI. Regarding the treatment history, 47/53 (89%) patients had failed at least one DMARDs therapy before the synovial biopsy. Specifically, 6/53 patients were DMARDs-naïve (11%), 46/53 previously failed at least one csDMARDs treatment line (87%), 20 (38%) have previously failed at least one b/tsDMARDs treatment line, of whom 17 failed TNF inhibitors (TNFis) and one failed a tsDMARD.

**Table 1:** Baseline characteristic of the 53 enrolled patients grouped by diagnosis.

Variables	Overall, N = 53	RA, N = 34	PsA, N = 19	p-value*^1^*
Female, *n (%)*	32 (60)	26 (76)	6 (32)	0.003
Age (years), *mean (SD)*	59 (11)	58 (12)	59 (9)	0.9
Weight (kg), *mean (SD)*	75 (16)	71 (16)	81 (13)	0.034
Disease duration (years), *mean (SD)*	12 (9)	11 (9)	12 (7)	0.9
Smoking exposure, *n (%)*	23 (43)	13 (38)	10 (53)	0.4
Fibromyalgia, *n (%)*	9 (17)	5 (15)	4 (21)	0.7
ACPA, *n (%)*	17 (32)	17 (50)	0 (0)	–
Rheumatoid factor, *n (%)*	20 (45)	20 (61)	0 (0)	–
Skin psoriasis, *n (%)*	15 (88)	0 (0)	15 (88)	–
CRP (mg/dl), *mean (SD)*	2.03 (2.77)	2.40 (3.10)	1.32 (1.92)	0.2
ESR (mm/h), *mean (SD)*	36 (26)	42 (25)	26 (25)	0.020
Moll and Wright subgroups				
Polyarthritis, *n (%)*	8 (42)	–	8 (42)	–
Oligoarthritis, *n (%)*	10 (53)	–	10 (53)	–
Predominant DIP joints involvement, *n (%)*	1 (5.3)	–	1 (5.3)	–
Arthritis mutilans, *n (%)*	0 (0)	–	0 (0)	–
Axial involvement, *n (%)*	0 (0)	–	0 (0)	–
Disease activity variables				
DAS28-CRP, *mean (SD)*	4.23 (0.95)	4.30 (0.98)	4.11 (0.92)	0.5
DAS28-ESR, *mean (SD)*	4.68 (1.07)	4.84 (1.03)	4.39 (1.10)	0.2
SDAI, *mean (SD)*	22 (10)	23 (11)	20 (7)	0.2
CDAI, *mean (SD)*	20.2 (8.7)	20.9 (10.0)	18.9 (5.4)	0.4
DAPSA, *mean (SD)*	24.65 (8.29)	–	24.65 (8.29)	–
HAQ (0-3), *mean (SD)*	0.91 (0.52)	0.96 (0.54)	0.84 (0.48)	0.4
VAS PhGA (0-100), *mean (SD)*	58 (17)	61 (15)	54 (20)	0.2
VAS PGA (0-100), *mean (SD)*	69 (17)	68 (15)	70 (21)	0.7
VAS Pain (0-100), *mean (SD)*	64 (18)	63 (15)	66 (23)	0.6
Tender joints count (0-68), *median (Q_1_-Q_3_)*	4 (2, 7)	4 (2, 7)	4 (3, 9)	0.5
Swollen joints count (0-66), *median (Q_1_-Q_3_)*	3 (2, 5)	3 (1, 5)	3 (2, 5)	0.9
GLOESS, *mean (SD)*	15 (13)	15 (13)	–	–
US and clinimetrics of joint to biopsied variables				
Grey scale synovitis (0-3), *median (Q_1_-Q_3_)*	2 (1, 2)	2 (1, 2)	2 (1, 3)	0.5
Joint effusion (0-3), *median (Q_1_-Q_3_)*	1 (1, 2)	1 (1, 2)	1 (1, 2)	>0.9
Power Doppler (0-3), *median (Q_1_-Q_3_)*	1 (0, 1)	1 (0, 1)	1 (0, 1)	0.8
Pain (0-100), *mean (SD)*	57 (28)	57 (26)	57 (31)	>0.9
Stiffness (0-100), *mean (SD)*	53 (28)	53 (27)	53 (30)	>0.9
Swelling (0-100), *mean (SD)*	56 (31)	54 (29)	58 (35)	0.7
Therapy variables				
DMARDs treatment näive	6 (11)	3 (8.8)	3 (16)	0.7
Previous methotrexate treatment	35 (66)	22 (65)	13 (68)	>0.9
Previous csDMARDs treatment	47 (89)	31 (91)	16 (84)	0.7
Previous b/tsDMARDs treatment	20 (38)	12 (35)	8 (42)	0.8
Previous TNFis treatment	17 (32)	9 (26)	8 (42)	0.4
Histopathological variables				
Krenn’s synovitis score (0-9), *median (Q_1_-Q_3_)*	6 (4, 6)	6 (4, 7)	4 (3, 6)	0.040
Krenn’s lining layer enlargement (0-3), *median (Q_1_-Q_3_)*	1 (1, 2)	1 (1, 2)	1 (1, 2)	0.3
Krenn’s resident cells density (0-3), *median (Q_1_-Q_3_)*	1 (1, 2)	1 (1, 2)	1 (1, 2)	0.2
Krenn’s inflammatory infiltrate (0-3), *median (Q_1_-Q_3_)*	2 (1, 3)	3 (2, 3)	2 (1, 2)	0.021

^1^Pearson’s Chi-squared test; Welch Two Sample t-test; Fisher’s exact test; Wilcoxon rank sum test.

RA, rheumatoid arthritis; PsA, psoriatic arthritis; ACPA, anti-citrullinated peptide antibody; DIP, distal interphalangeal joints; CRP, C- reactive protein; ESR, erythrocyte sedimentation rate; DAS, disease activity score; SDAI, simplex disease activity index; CDAI, clinical disease activity index; HAQ, health assessment questionnaire; VAS, visual analogue scale; PhGA, physician global assessment; PGA patient global assessment; GLOESS, global OMERACT-EULAR score system; US, ultrasound; csDMARD, conventional synthetic disease modifying antirheumatic drugs; b/tsDMARD, biological/targeted synthetic disease modifying antirheumatic drugs; TNFis, tumour necrosis factor inhibitors.

### 3.2 Histological assessment of synovial samples

**Figure 1:**
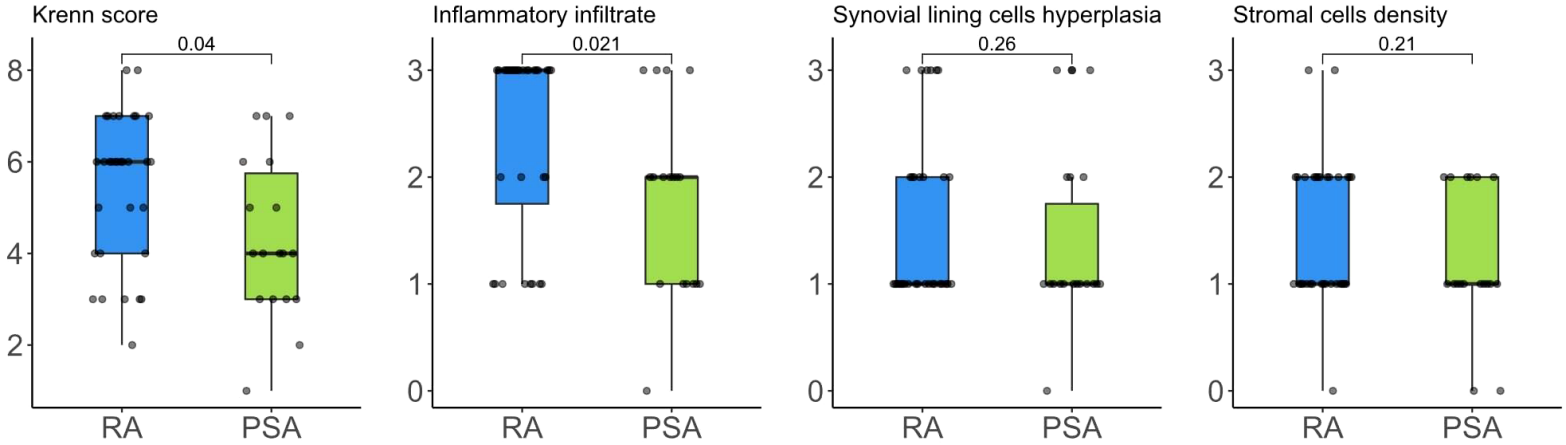
Krenn’s synovitis score and its components distribution in RA e PsA patients.

KSS was calculated for 50 patients (3 synovial samples were judged inadequate for histological examination). The median KSS was 6 (Q1-Q3 4-6) and high-grade synovitis was found in 30/50 (60%) patients, with higher frequency in RA over PsA patients (RA 67.6% vs PsA 36.8%, p=0.047). Moreover, RA patients showed a higher score in inflammatory infiltrates component (p=0.021) (**Figure 1**).

### 3.3 Association between high grade Krenn’s Synovitis Score and baseline clinical variables

In univariable logistic regression analysis, ESR (1 mm/h OR 1.04, 95%CI 1.01-1.08), RA diagnosis (OR 4.02, 95%CI 1.22-14.30), DAS28-ESR (OR 2.23, 95%CI 1.16-5.15) associated with higher probability of high-grade KSS at the histological evaluation (**Table 2**). The detection of positive Power Doppler (PD) signal (OR 3.55, 95%CI 1.08-12.78) and moderate to high synovial effusion (OR 7.56, 95%CI 1.99-37.95) at ultrasound examination of the joint to be biopsied were positively associated to KSS≥5, as well as a greater patient-reported stiffness in the joint to be biopsied (OR 1.03, 95%CI 1.01-1.06). In multivariable analysis, the stepwise backward selection of variables found a stronger association for the ultrasound synovial effusion (OR 9.26, 95%CI 2.12-53.91) and ESR (1 mm/h OR 1.04, 95%CI 1.01-1.08).

**Table 2:** Univariable logistic regression analysis for association between high grade synovitis (KSS≥5) and baseline clinical variables.

Variables		KSS<5	KSS≥5	OR (95%CI, p-value)		
				Univariable	Multivariable	
Gender, *n (%)*	M	10 (52.6)	9 (47.4)	-	-
	F	10 (32.3)	21 (67.7)	2.33 (0.73-7.76, p=0.157)	-
Age, *mean (SD)*		59.3 (10.7)	58.5 (10.9)	0.99 (0.94-1.05, p=0.806)	-
Disease duration, *mean (SD)*		10.2 (6.7)	12.2 (9.7)	1.03 (0.96-1.11, p=0.438)	-
Weight, *mean (SD)*		78.9 (16.0)	72.2 (15.6)	0.97 (0.93-1.01, p=0.154)	-
Smoking exposure, *n (%)*	No	11 (37.9)	18 (62.1)	-	-
	Yes	9 (42.9)	12 (57.1)	0.81 (0.26-2.58, p=0.726)	-
Fibromyalgia, *n (%)*	No	14 (34.1)	27 (65.9)	-	-
	Yes	6 (66.7)	3 (33.3)	0.26 (0.05-1.14, p=0.084)	-
CRP (mg/dl), *mean (SD)*		1.5 (2.2)	2.5 (3.1)	1.17 (0.93-1.58, p=0.248)	-
ESR (mm/h), *mean (SD)*		23.7 (21.6)	43.9 (25.7)	1.04 (1.01-1.08, p=0.013)	1.04 (1.01-1.08, p=0.012)
Diagnosis, *n (%)*	PSA	11 (61.1)	7 (38.9)	-	-
	RA	9 (28.1)	23 (71.9)	4.02 (1.22-14.30, p=0.026)	-
HAQ (0-3), *mean (SD)*		0.8 (0.6)	1.0 (0.5)	1.46 (0.48-4.81, p=0.506)	-
VAS Pain (0-100), *mean (SD)*		68.6 (20.4)	61.7 (16.9)	0.98 (0.94-1.01, p=0.209)	-
VAS PhGA (0-100), *mean (SD)*		56.2 (20.9)	60.3 (15.2)	1.01 (0.98-1.05, p=0.421)	-
VAS PGA (0-100), *mean (SD)*		73.2 (20.9)	66.5 (14.0)	0.98 (0.94-1.01, p=0.179)	-
Tender joints count (0-68), *median (Q_1_-Q_3_)*		4 (3, 9)	4 (2, 7)	0.98 (0.87-1.10, p=0.725)	-
Swollen joints count (0-66), *median (Q_1_-Q_3_)*		3 (2, 6)	3 (2, 4)	1.01 (0.88-1.19, p=0.856)	-
DAS28-CRP, *mean (SD)*		4.1 (0.9)	4.4 (1.0)	1.35 (0.73-2.78, p=0.364)	-
DAS28-ESR, *mean (SD)*		4.3 (1.1)	5.0 (1.0)	2.23 (1.16-5.15, p=0.032)	-
SDAI, *mean (SD)*		21.6 (7.7)	23.4 (11.1)	1.02 (0.96-1.10, p=0.540)	-
CDAI, *mean (SD)*		20.1 (6.5)	20.9 (10.2)	1.01 (0.95-1.09, p=0.752)	-
US and clinimetrics variables of the joint to biopsied						
Stiffness (0-100), *mean (SD)*		38.9 (29.7)	60.0 (24.2)	1.03 (1.01-1.06, p=0.015)	-
Pain (0-100), *mean (SD)*		54.5 (30.3)	59.5 (25.9)	1.01 (0.99-1.03, p=0.530)	-
Swelling (0-100), *mean (SD)*		44.2 (35.6)	61.3 (25.7)	1.02 (1.00-1.04, p=0.062)	-
Grey scale synovitis ≥2, *n (%)*	No	10 (52.6)	9 (47.4)	-	-
	Yes	9 (31.0)	20 (69.0)	2.47 (0.76-8.42, p=0.138)	-
Joint effusion≥2, *n (%)*	No	16 (57.1)	12 (42.9)	-	-
	Yes	3 (15.0)	17 (85.0)	7.56 (1.99-37.95, p=0.006)	9.26 (2.12-53.91, p=0.006)
Power Doppler ≥1, *n (%)*	No	13 (54.2)	11 (45.8)	-	-
	Yes	6 (25.0)	18 (75.0)	3.55 (1.08-12.78, p=0.043)	-

KSS, Krenn’s synovitis score; RA, rheumatoid arthritis; PsA, psoriatic arthritis; CRP, C reactive protein; ESR, erythrocyte sedimentation rate; DAS, disease activity score; SDAI, simplex disease activity index; CDAI, clinical disease activity index; HAQ, health assessment questionnaire; VAS, visual analogue scale; PhGA, physician global assessment; PGA patient global assessment; US, ultrasound.

### 3.3 Association between high Krenn’s inflammatory infiltrate and baseline clinical variables

We assessed the association between high Krenn’s inflammatory infiltrates (score =3) and clinical variables in the univariable logistic regression analysis, showing a positive relationship for female gender (OR 4.43, 95%CI 1.33-16.77), ESR (OR 1.03, 95%CI 1.01-1.06), RA diagnosis (OR 5.83, 95%CI 1.66-24.52), DAS28-ESR (OR 2.03, 95%CI 1.09-4.39), joint stiffness (OR 1.03, 95%CI 1.00-1.05) and positive PD signal in the joint to be biopsied (OR 3.33, 95%CI 1.05-11.39) (**Table 3**). When applying the backward stepwise method, the variables included in the multivariable analysis were RA diagnosis (OR 7.98, 95%CI 2.02-39.53) and joint stiffness (OR 1.03, 95%CI 1.01-1.06).

**Table 3:** Univariable logistic regression analysis for association between high Krenn inflammatory infiltrates score (=3) and baseline clinical variables.

Variables		Krenn’s infiltrates <3	Krenn’s infiltrates =3	OR (95%CI, p-value)	
				Univariable	Multivariable
Gender, *n (%)*	M	14 (73.7)	5 (26.3)	-	-
	F	12 (38.7)	19 (61.3)	4.43 (1.33-16.77, p=0.020)	-
Age, *mean (SD)*		59.7 (11.7)	57.9 (9.7)	0.98 (0.93-1.04, p=0.540)	-
Disease duration, *mean (SD)*		11.9 (8.4)	10.9 (9.0)	0.99 (0.92-1.05, p=0.673)	-
Weight, *mean (SD)*		78.2 (14.8)	71.3 (16.7)	0.97 (0.93-1.01, p=0.134)	-
Smoking exposure, *n (%)*	0	15 (51.7)	14 (48.3)	-	-
	1	11 (52.4)	10 (47.6)	0.97 (0.31-3.02, p=0.963)	-
Fibromyalgia, *n (%)*	0	20 (48.8)	21 (51.2)	-	-
	1	6 (66.7)	3 (33.3)	0.48 (0.09-2.07, p=0.337)	-
CRP (mg/dl), *mean (SD)*		1.8 (2.4)	2.5 (3.2)	1.11 (0.90-1.42, p=0.341)	-
ESR (mm/h), *mean (SD)*		27.1 (23.9)	45.6 (25.0)	1.03 (1.01-1.06, p=0.019)	-
Diagnosis, *n (%)*	PSA	14 (77.8)	4 (22.2)	-	-
	RA	12 (37.5)	20 (62.5)	5.83 (1.66-24.52, p=0.009)	7.98 (2.02-39.53, p=0.005)
HAQ (0-3), *mean (SD)*		0.9 (0.6)	1.0 (0.5)	1.48 (0.50-4.62, p=0.483)	-
VAS Pain (0-100), *mean (SD)*		67.7 (19.0)	61.2 (17.8)	0.98 (0.95-1.01, p=0.232)	-
VAS PhGA (0-100), *mean (SD)*		57.1 (20.0)	60.4 (14.9)	1.01 (0.98-1.05, p=0.506)	-
VAS PGA (0-100), *mean (SD)*		72.5 (19.8)	65.6 (13.5)	0.98 (0.94-1.01, p=0.164)	-
Tender joints count (0-68), *median (Q_1_-Q_3_)*		4 (3, 8)	4 (3, 8)	1.01 (0.90-1.13, p=0.927)	-
Swollen joints count (0-66), *median (Q_1_-Q_3_)*		3 (2, 6)	3 (2, 6)	1.04 (0.90-1.21, p=0.603)	-
DAS28-CRP, *mean (SD)*		4.2 (1.0)	4.4 (0.9)	1.36 (0.75-2.71, p=0.330)	-
DAS28-ESR, *mean (SD)*		4.4 (1.1)	5.1 (1.0)	2.03 (1.09-4.39, p=0.042)	-
SDAI, *mean (SD)*		21.6 (7.7)	23.8 (11.8)	1.02 (0.97-1.10, p=0.456)	-
CDAI, *mean (SD)*		19.9 (6.2)	21.2 (11.1)	1.02 (0.95-1.10, p=0.592)	-
*US and clinimetric variables of the joint to biopsied*					
Stiffness (0-100), *mean (SD)*		42.8 (30.3)	61.2 (22.8)	1.03 (1.00-1.05, p=0.027)	1.03 (1.01-1.06, p=0.022)
Pain (0-100), *mean (SD)*		56.4 (30.0)	58.8 (25.2)	1.00 (0.98-1.02, p=0.763)	-
Swelling (0-100), *mean (SD)*		51.6 (34.7)	57.9 (26.4)	1.01 (0.99-1.03, p=0.470)	-
Grey scale synovitis ≥2, *n (%)*	0	12 (63.2)	7 (36.8)	-	-
	1	13 (44.8)	16 (55.2)	2.11 (0.66-7.17, p=0.217)	-
Joint effusion≥2, *n (%)*	0	17 (60.7)	11 (39.3)	-	-
	1	8 (40.0)	12 (60.0)	2.32 (0.73-7.75, p=0.160)	-
Power Doppler ≥1, *n (%)*	0	16 (66.7)	8 (33.3)	-	-
	1	9 (37.5)	15 (62.5)	3.33 (1.05-11.39, p=0.046)	-

RA, rheumatoid arthritis; PsA, psoriatic arthritis; CRP, C reactive protein; ESR, erythrocyte sedimentation rate; DAS, disease activity score; SDAI, simplex disease activity index; CDAI, clinical disease activity index; HAQ, health assessment questionnaire; VAS, visual analogue scale; PhGA, physician global assessment; PGA patient global assessment; US, ultrasound.

### 3.4 Role of histological features in predicting response to therapy

After the synovial biopsy, 38/53 (71.7%) patients started a new b/tsDMARDs therapy and, within these, 6/38 were treated with a Janus Kinases inhibitor (JAKi). The other patients increased the ongoing DMARDs therapy (5/53, 9.4%), started a new csDMARDs (3/53, 5.7%) or were treated with other therapies (i.e., steroid injections, NSAIDs). The patterns of treatment did not significantly differ between patients with high-grade and low-grade synovitis (**Table 4**).

**Table 4:** Comparison of treatment patterns in patients with high-grade and low-grade synovitis.

Therapy	Overall, N = 50	KSS<5, N = 20	KSS≥5, N = 30	p-value*^1^*
Switch to new csDMARDs, *n (%)*	3 (6.0)	0 (0)	3 (10)	0.3
Switch to new b/tsDMARDs, *n (%)*	31 (62)	13 (65)	18 (60)	>0.9
Switch to new tsDMARDs, *n (%)*	6 (12)	1 (5.0)	5 (17)	0.4
Increased ongoing DMARDs, *n (%)*	3 (6.0)	2 (10)	1 (3.3)	0.6
Others therapies, *n (%)*	7 (14)	4 (20)	3 (10)	0.4

^1^Fisher’s exact test; Pearson’s Chi-squared test.

csDMARD, conventional synthetic disease modifying antirheumatic drugs; bDMARD, biological disease modifying antirheumatic drugs; tsDMARD, targeted synthetic disease modifying antirheumatic drugs.

At 24 weeks visit, 24/51 (47.1%) patients achieved DAS28-CRP remission, 22/53 (41.5%) ACR20 response, 17/53 (32.1%) ACR50 response and 18/51 (35.3%) Boolean2.0 remission. CRP values at 24 weeks were not available for 2 patients, therefore DAS28 remission and Boolean2.0 criteria were not calculated. Considering PsA patients individually, 5/19 (26,32%) were in remission according to DAPSA (≤4) and 13/19 (68,42%) in DAPSA low disease activity (≤14).

**Figure 2:**
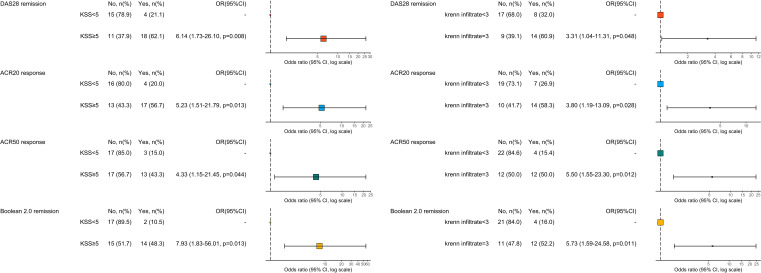
Univariable logistic regression analysis for prediction of response/remission defined by ACR20 response criteria, ACR50 response criteria, Boolean 2.0 remission criteria and DAS28-CRP remission. *OR*>1 means favour to response, *OR*<1 means against response. ACR20 and ACR50, American College of Rheumatology response criteria; DAS, disease activity score; KSS, Krenn’s synovitis score; OR, odds ratio.

The results of the univariable logistic regression analysis are shown in **Figure 2**. High-grade KSS significantly associated with higher probability of achieving DAS28-CRP remission (OR 6.14, 95%CI 1.73-26.10), ACR20 response (OR 5.23, 95%CI 1.51-21.79), ACR50 response (OR 4.33, 95%CI 1.15-21.45) and Boolean2.0 remission (OR 7.93, 95%CI 1.83-56.01) criteria (**Figure 2**). The presence of Krenn’s inflammatory infiltrates score equal to 3 similarly revealed a predictive role in achieving the targets (DAS28-CRP OR 3.31, 95%CI 1.04-11.31, ACR20 3.80, 1.19-13.09, ACR50 5.50, 1.55-23.30, Boolean2.0 remission 5.73, 1.59-24.58). After adjusting the models for diagnosis, the association between high-grade KSS or high Krenn’s inflammatory infiltrates and clinical response to therapy were confirmed independently from diagnosis, apart for the association between high Krenn’s inflammatory infiltrates and DAS28-CRP remission which was not significant (OR 3.36, 95%CI 0.95-13.17, p=0.067) (**Supplementary Table S2**-**S3**). Contrariwise, baseline ultrasound features taken individually (positive power Doppler signal, grey scale synovitis score greater than 1 and joint effusions score greater than 1) were not predictive of response and, similarly, baseline clinical variables did not predict the most stringent definitions of response, despite associating with ACR20 (**Supplementary Table S4**).

## 4 Discussion

Among the procedures to be included in the histological reporting of synovial biopsies, semiquantitative scores are suggested, but the clinical role of such scores is still under-recognized and under-reported (30). Here, we investigated the clinical and instrumental determinants of KSS in a prospective cohort of IAs patients, highlighting that several markers of systemic and local inflammation associate with high-grade synovitis, such as ESR, disease activity, PD-positive synovitis, joint effusion and joint stiffness, as well as RA Vs PsA diagnosis. Moreover, we confirmed a predictive role of high-grade KSS and high-grade synovial inflammatory infiltrate score in antedating clinical response to treatment.

Data regarding the clinical determinants of the KSS score are scarce in the literature. In the present study, we demonstrated that pro-inflammatory markers, like ESR, DAS28-ESR, PD-positive synovitis and synovial effusion, associate with high-grade synovitis in IAs. When we focused on the clinical determinants of high Krenn’s inflammatory infiltrates, we demonstrated a significant association with female gender, ESR, DAS28-ESR, joint stiffness and PD-positive synovitis. This partially confirms the available literature data. In patients with long-standing RA (mean disease duration 13.1 years) who underwent isolated synoviectomy or total joint replacement plus synoviectomy or arthrodesis, the KSS reflected disease activity defined by CDAI (31). The same result was obtained in a prospective cohort study with 545 RA and 167 PsA patients, in which DAS28 directly associated with KSS in patients with RA and PsA, both naïve and refractory to treatment with DMARDs (32). Furthermore, DMARDs-naïve RA patients with a disease duration of less than three months had a lower KSS than those with a longer disease duration, but KSS was globally higher in DMARDs-naïve RA than in RA resistant to csDMARDs or in remitting patients. Again, the PD-score in the biopsied joint directly correlated with KSS in both RA and PsA patients (32). In another dataset of patients with early and long-standing RA, the KSS correlated with both the EULAR-OMERACT ultrasound combined score and with the RAMRIS MRI synovitis score (48). However, no compelling association has been already confirmed, suggesting that the informative potential of KSS could not be easily inferred from ultrasound or clinical features. In our cohort of patients with active disease, RA patients had higher median KSS and Krenn’s inflammatory infiltrates than PsA patients; this was not confirmed by other authors (49). With respect to the higher prevalence of female gender among high inflammatory infiltrates scores, the notion of a worse course of the disease in females with RA and PsA is not new (50, 51). Our findings agree with the notion that, whichever inflammatory pathways are involved, high grade synovitis is the result of an active disease, independently from diagnosis. This concept, worthy of further investigation, is partially innovative for rheumatology. Based on this assumption, we could hypothesize that high-grade chronic synovitis, as an expression of the target organ involvement, is transversal to disease classification, and focusing on KSS might serve as a useful information tool for disease stratification especially in the prognostic phase and in prediction of the therapeutic response (52).

Specifically, the role of KSS in predicting response to therapy is debated. The work by Alivernini et al. showed that the presence of a low-grade KSS at baseline predicts a higher response rate to DMARDs (defined as DAS28 remission at 6 months) in treatment-naive RA patients (32). In the R4RA randomized trial, the KSS was not able to discriminate patients who were responders to either tocilizumab or rituximab after the failure of at least one bDMARD (22). In a different and more variegated population, we demonstrated that a high KSS associated with response to therapy according to several definitions, like DAS28-CRP remission, Boolean2.0 remission, ACR20 and ACR50. This was demonstrated also for high Krenn’s inflammatory infiltrates, and independently from diagnosis. We analyzed the single components of the ACR20 response criteria, and none emerged over the others in justifying the absence of response (**Supplementary Table S5**), so we can summarize that disease activity still persisted in this group of patients. Thus, we hypothesize that, in patients with high-grade KSS, several treatment paradigms (cs/b/tsDMARDs) might permit the achievement of the treatment target, while for patients with low-grade synovitis this remains more difficult. From a speculative point of view, it is possible that high-grade KSS is characterized by a greater involvement of pro-inflammatory pathways, susceptible to modification by the drugs currently approved for the treatment of RA and PsA, resulting in a greater probability of therapeutic response. Conversely, low-grade KSS might involve different pathogenic mechanisms, such as fibrosis, stromal activation and chronic pain phenomena, less susceptible to response via inhibition of the pathways interceptable with the currently available therapies. And this was confirmed, at least partially, with the definition of the ‘synovial pathotypes’ concept. After the initial definition of Dennis et al. derived from patients with long standing RA undergoing arthroplasty or synoviectomy (16), the systematic application of this concept using immunohistochemistry (IHC) to an early RA population (Pathobiology of Early Arthritis Cohort, PEAC) undergoing ultrasound-guided synovial biopsy led to the definition of the ‘diffuse myeloid’, ‘lymphomyeloid’ and ‘pauci-immune’ pathotypes (17–19). This concept was also explored in populations with RA refractory to csDMARDs (21, 53). Studies assessing the prognostic role of synovial pathotypes suggest that TNF-dependent inflammatory pathways are involved in the activation of fibroblast-like synoviocytes (FLS) and enhancement of T cells/FLS interactions, mostly evident in lymphoid pathotypes (23). Myeloid- and lymphoid-associated gene signatures associate with response to rituximab or tocilizumab (20), while, on the other hand, a pauci-immune-fibroid pathotype, which is characterized by low immune cells infiltration, associated with worse response to csDMARDs and TNFis (18, 21). With well-defined treatment schedules for patients with early RA and PsA, and in presence of an acceptable cost-effectiveness profile of first-line methotrexate (MTX) boundary for both the pathologies, a concept like the one explored in our study might apply more appropriately to difficult-to-treat diseases (54, 55). Here, the characterization of the synovial tissue should inform on whether the disease is still supported by a pro-inflammatory movens (e.g. high-grade synovitis) that could be targeted by one of the available therapies, included JAKis, or, conversely, by other non-inflammatory pathways which could belong to low-grade synovitis spectrum (56). Specifically, it remains actually not known the best treatment strategy to adopt for patients with refractory RA deemed to be ‘non-inflammatory’ (NIRRA). Since a treatment-delay for first-line MTX start does not impact on the future development of NIRRA, while its very-early adoption prevents the persistent-inflammatory refractory RA (PIRRA) onset (57), the possibility arising is that a niches of patients with a different low-inflammatory disease exists, and a minimally-invasive procedure like ultrasound-guided synovial biopsy may help to disease stratification (58).

To this end, the role of synovial tissue analysis in translational research is rapidly arising, in particular for new drugs development, drugs repurposing, biomarkers discovery. However, several barriers still limit its complete clinical adoption in patients with IAs (12, 14). With regard to histological scores, EULAR and OMERACT advocate the adoption of a synovitis score, like KSS, as part of the reporting of synovial biopsies in synovial tissue research (30). However, few studies still semi-quantitatively evaluated the features of synovial inflammation in IAs, and this applies particularly to PsA. A recent systematic literature review (SLR) reported that none of the studies assessing the synovial effects of approved b/tsDMARDs in PsA adopted the KSS (13). Among the recently-released EULAR points to consider for minimal reporting requirements in synovial tissue research in rheumatology (59), the adoption of well-categorized scores is advocated, since the available literature rarely describes the scoring systems adopted, with several chains of references to previous publications, but not to the original scoring system. This should help in generalizing and validating the outcomes described. Moreover, the EULAR task force suggested that patient disease activity measures or disease stage should be described, since only 62% of the studies included in the SLR reported clinical data, such as disease activity and current therapy (59). Here, we wanted to focus on a population of patients with active IAs, at the time of treatment modification, with a median KSS score correspondent to high-grade synovitis, in order to analyze in detail the role of the KSS to be practically used in the clinics. Secondly, we decided to focus on both RA and PsA patients with peripheral synovitis, since these diseases share common pathogenic pathways that result in similar treatment algorithms and types of drugs. The available literature regarding the contact points between RA and PsA synovitis focused mostly on the differences, highlighting higher values of neovascularization markers, CD163^pos^ macrophages and CD117^pos^/c-Kit^pos^ mast cells in PsA, with more enlarged lining layer thickness in RA (60–63). However, the majority of synovial studies frequently reported no significant variations in the number and types of cell infiltrates or in the distribution of inflammatory mediators in RA Vs PsA. As recently suggested, a continuum could exist from seropositive RA to seronegative RA and PsA, with comparable CD68^pos^ macrophages and CD3^pos^ T lymphocytes counts, but with higher CD20^pos^ B-cells scores in seropositive RA Vs seronegative RA and PsA (49), and higher CD138^pos^ plasma cells in seronegative RA Vs PsA (64). This justifies the combined analysis of the synovitis score in patients with different diseases (closer-than-expected), who are experiencing an upgrade in similar treatment schedules.

This study has some limitations that should be mentioned. What emerged from this work represents an analysis of a single-center cohort study, with limited sample size. Furthermore, we did not assess the synovial pathotype or the bulk-tissue mRNA expression, which were out of the scope of this study. Other limitations include the lack of external validation in a different cohort, the absence of joint radiographic assessment data, the estimation of KSS by a single pathologist, and the under-representation of non-Caucasian patients. We focused on PsA patients with peripheral synovitis, thus our results cannot be generalizable to the whole PsA disease spectrum (65), and, similarly, no meaningful information can be provided with respect to specific peripheral PsA subsets, like oligoarticular or polyarticular, which share some histological similarities and differences (49, 62). Finally, no robust information is inferable regarding the single mechanisms of action of DMARDs, since we decided to focus on the treatment modification strategy as a whole. However, our study reasonably reflects a population of chronic IA patients and the clinical heterogeneity of presentation of diseases. Moreover, the applicability of an inexpensive score obtained following a minimally invasive well-tolerated procedure represents an advantageous aspect in terms of reproducibility and potential clinical applicability, as it is expected that ultrasound-guided synovial biopsy procedures may spread to several centers, following training programs and research collaborations (66, 67).

In conclusion, we demonstrated that a simple tool like KSS might help in prognostic characterization of chronic IA patients, and high-grade synovitis represents an expression of pro-inflammatory pathways that appear to be interceptable by currently approved treatment algorithms.

## Data availability statement

The raw data supporting the conclusions of this article will be made available by the authors, without undue reservation.

## Ethics statement

The studies involving humans were approved by Comitato Etico Area Vasta Emilia Centro della Regione Emilia-Romagna (CE-AVEC). The studies were conducted in accordance with the local legislation and institutional requirements. The participants provided their written informed consent to participate in this study.

## Author contributions

CG: Conceptualization, Data curation, Formal analysis, Investigation, Methodology, Project administration, Writing – original draft. MT: Data curation, Investigation, Writing – review & editing. IC: Data curation, Investigation, Writing – review & editing. GL: Conceptualization, Data curation, Methodology, Writing – review & editing. AB: Data curation, Investigation, Supervision, Writing – review & editing. CS: Conceptualization, Data curation, Funding acquisition, Investigation, Methodology, Project administration, Supervision, Validation, Writing – review & editing. MG: Conceptualization, Data curation, Investigation, Project administration, Resources, Supervision, Writing – review & editing. ES: Conceptualization, Data curation, Formal analysis, Investigation, Methodology, Project administration, Resources, Supervision, Validation, Visualization, Writing – original draft.
