# Supplementary material for: High-grade synovitis associates with clinical markers and response to therapy in chronic inflammatory arthritis: post hoc analysis of a synovial biomarkers prospective cohort study
Source: Front Immunol. 2024 Jan 11;14:1298583. doi: 10.3389/fimmu.2023.1298583 (PMC10808827; doi:10.3389/fimmu.2023.1298583)
Supplement: Supplementary file 1 [file DataSheet_1.pdf]

Supplementary Materials

Table S1. Histopathological Krenn's synovitis score (1).

Score	*Definition*
Enlargement of the synovial lining cell layer	
0 points	The lining cells form one layer.
1 point	The lining cells form 2–3 layers.
2 points	The lining cells form 4–5 layers, few multinucleated cells might occur.
3 points	The lining cells form more than 5 layers, the lining might be ulcerated and multinucleated cells might occur.
Density of the resident cells	
0 points	The synovial stroma shows normal cellularity.
1 point	The cellularity is slightly increased.
2 points	The cellularity is moderately increased, multinucleated cells might occur.
3 points	The cellularity is greatly increased, multinucleated giant cells, pannus formation and rheumatoid granulomas might occur.
Inflammatory infiltrate	
0 points	No inflammatory infiltrate.
1 point	Few mostly perivascular situated lymphocytes or plasma-cells.
2 points	Numerous lymphocytes or plasma-cells, sometimes forming follicle-like aggregates.
3 points	Dense band-like inflammatory infiltrate or numerous large follicle-like aggregates.
Cumulative score	
0-1 points	No synovitis
2-4 points	Low-grade synovitis
5-9 points	High-grade synovitis

1. Krenn V, Morawietz L, Burmester G-R, Kinne RW, Mueller-Ladner U, Muller B, Haupl T. Synovitis score: discrimination between chronic low-grade and high-grade synovitis. *Histopathology* (2006) 49:358–364. doi: 10.1111/j.1365-2559.2006.02508.x

Table S2. Univariable and multivariable logistic regression analysis for association between response to therapy at 24 weeks and high-grade synovitis (KSS >=5).

**DAS28 remission**		**No**, n (%)	**Yes**, n (%)	**OR (univariable)**[1]	**OR (adjusted for diagnosis)**[1]
	KSS<5	15 (78.9)	4 (21.1)	-	-
	KSS ≥5	11 (37.9)	18 (62.1)	6.14 (1.73-26.10, p=0.008)	6.52 (1.69-31.29, p=0.010)

**ACR20**		**No**, n (%)	**Yes**, n (%)	**OR (univariable)**[1]	**OR (adjusted for diagnosis)**[1]
	KSS<5	16 (80.0)	4 (20.0)	-	-
	KSS ≥5	13 (43.3)	17 (56.7)	5.23 (1.51-21.79, p=0.013)	7.60 (1.88-42.03, p=0.009)

**ACR50**		**No**, n (%)	**Yes**, n (%)	**OR (univariable)**[1]	**OR (adjusted for diagnosis)**[1]
	KSS<5	17 (85.0)	3 (15.0)	-	-
	KSS ≥5	17 (56.7)	13 (43.3)	4.33 (1.15-21.45, p=0.044)	5.58 (1.32-32.21, p=0.031)

**Boolean2.0**		**No**, n (%)	**Yes**, n (%)	**OR (univariable)**[1]	**OR (adjusted for diagnosis)**[1]
	KSS<5	17 (89.5)	2 (10.5)	-	-
	KSS ≥5	15 (51.7)	14 (48.3)	7.93 (1.83-56.01, p=0.013)	8.73 (1.83-67.60, p=0.014)

[1]Odds ratio (95%CI, p-value)

Abbreviations: ACR20 and ACR50, American College of Rheumatology response criteria; DAS, disease activity score; KSS, Krenn's synovitis score; OR, odds ratio.

Table S3. Univariable and multivariable logistic regression analysis for association between response to therapy at 24 weeks and Krenn's inflammatory infiltrate score equal to 3.

**DAS28 remission**		**No**, n (%)	**Yes**, n (%)	**OR (univariable)**[1]	**OR (adjusted for diagnosis)**[1]
	Krenn's infiltrates<3	17 (68.0)	8 (32.0)	-	-
	Krenn's infiltrates=3	9 (39.1)	14 (60.9)	3.31 (1.04-11.31, p=0.048)	3.36 (0.95-13.17, p=0.067)
**ACR20**		**No**, n (%)	**Yes**, n (%)	**OR (univariable)**[1]	**OR (adjusted for diagnosis)**[1]
	Krenn's infiltrates<3	19 (73.1)	7 (26.9)	-	-
	Krenn's infiltrates=3	10 (41.7)	14 (58.3)	3.80 (1.19-13.09, p=0.028)	5.87 (1.53-29.43, p=0.016)
**ACR50**		**No**, n (%)	**Yes**, n (%)	**OR (univariable)**[1]	**OR (adjusted for diagnosis)**[1]
	Krenn's infiltrates<3	22 (84.6)	4 (15.4)	-	-
	Krenn's infiltrates=3	12 (50.0)	12 (50.0)	5.50 (1.55-23.30, p=0.012)	9.82 (2.12-73.77, p=0.009)
**Boolean2.0**		**No**, n (%)	**Yes**, n (%)	**OR (univariable)**[1]	**OR (adjusted for diagnosis)**[1]
	Krenn's infiltrates<3	21 (84.0)	4 (16.0)	-	-
	Krenn's infiltrates=3	11 (47.8)	12 (52.2)	5.73 (1.59-24.58, p=0.011)	6.83 (1.63-37.73, p=0.014)

[1]Odds ratio (95%CI, p-value)

Abbreviations: ACR20, American College of Rheumatology response criteria; DAS, disease activity score; OR, odds ratio.

Table S4 – Univariable logistic regression analysis for the association between response and baseline US features.

**DAS28 remission**		**No,** n(%)	**Yes,** n(%)	**OR (95%CI, p-value)**
Power Doppler ≥1	0	15 (65.2)	8 (34.8)	-
	1	12 (46.2)	14 (53.8)	2.19 (0.70-7.17, p=0.184)
Joint effusion ≥2	0	16 (59.3)	11 (40.7)	-
	1	11 (50.0)	11 (50.0)	1.45 (0.47-4.60, p=0.518)
Grey scale synovitis ≥2	0	11 (57.9)	8 (42.1)	-
	1	16 (53.3)	14 (46.7)	1.20 (0.38-3.92, p=0.755)
**ACR20 response**		**No,** n(%)	**Yes,** n(%)	**OR (95%CI, p-value)**
Power Doppler ≥1	0	15 (60.0)	10 (40.0)	-
	1	16 (61.5)	10 (38.5)	0.94 (0.30-2.91, p=0.910)
Joint effusion ≥2	0	18 (62.1)	11 (37.9)	-
	1	13 (59.1)	9 (40.9)	1.13 (0.36-3.54, p=0.829)
Grey scale synovitis ≥2	0	13 (68.4)	6 (31.6)	-
	1	18 (56.2)	14 (43.8)	1.69 (0.52-5.84, p=0.391)
**ACR50 response**		**No,** n(%)	**Yes,** n(%)	**OR (95%CI, p-value)**
Power Doppler ≥1	0	18 (72.0)	7 (28.0)	-
	1	18 (69.2)	8 (30.8)	1.14 (0.34-3.91, p=0.828)
Joint effusion ≥2	0	20 (69.0)	9 (31.0)	-
	1	16 (72.7)	6 (27.3)	0.83 (0.24-2.81, p=0.770)
Grey scale synovitis ≥2	0	15 (78.9)	4 (21.1)	-
	1	21 (65.6)	11 (34.4)	1.96 (0.55-8.18, p=0.317)
**Boolean2.0 remission**		**No,** n(%)	**Yes,** n(%)	**OR (95%CI, p-value)**
Power Doppler ≥1	0	18 (78.3)	5 (21.7)	-
	1	15 (57.7)	11 (42.3)	2.64 (0.78-9.99, p=0.131)
Joint effusion ≥2	0	19 (70.4)	8 (29.6)	-
	1	14 (63.6)	8 (36.4)	1.36 (0.41-4.57, p=0.618)
Grey scale synovitis ≥2	0	14 (73.7)	5 (26.3)	-
	1	19 (63.3)	11 (36.7)	1.62 (0.47-6.12, p=0.453)

Abbreviations: ACR20 and ACR50, American College of Rheumatology response criteria; DAS, disease activity score; OR, odds ratio.

Table S5. Clinical and laboratory data collected at 24 weeks visit, grouped by ACR20 response achievement.

	**ACR20 not responder**, N = 31	**ACR20 responder**, N = 22	**p-value**[1]
Tender joints count (0-68), *median (Q_1-Q_3)*	1 (0, 5)	1 (0, 1)	0.028
Swollen joints count (0-66), *median (Q_1-Q_3)*	2 (1, 3)	1 (0, 2)	0.015
HAQ (0-3), *mean (SD)*	0.84 (0.64)	0.43 (0.42)	0.015
VAS PhGA (0-100), *mean (SD)*	41 (26)	15 (13)	<0.001
VAS PGA (0-100), *mean (SD)*	48 (31)	16 (15)	<0.001
VAS Pain (0-100), *mean (SD)*	47 (33)	15 (16)	<0.001
CRP (mg/dl), *mean (SD)*	0.67 (1.30)	0.53 (0.51)	0.2
ESR (mm/h), *mean (SD)*	17 (18)	19 (14)	0.3

[1] Wilcoxon rank sum test

Abbreviations: ACR20, American College of Rheumatology response criteria; HAQ, health assessment questionnaire; VAS, visual analogue scale; PhGA, physician global assessment; PGA patient global assessment; CRP, C-reactive protein; ESR, erythrocyte sedimentation rate.
